# Folate Receptor Targeted Alpha-Therapy Using Terbium-149

**DOI:** 10.3390/ph7030353

**Published:** 2014-03-13

**Authors:** Cristina Müller, Josefine Reber, Stephanie Haller, Holger Dorrer, Ulli Köster, Karl Johnston, Konstantin Zhernosekov, Andreas Türler, Roger Schibli

**Affiliations:** 1 Center for Radiopharmaceutical Sciences ETH-PSI-USZ, Paul Scherrer Institute, 5232 Villigen-PSI, Switzerland; E-Mails: josefine.reber@gmail.com (J.R.); stephanie.haller@psi.ch (S.H.); roger.schibli@psi.ch (R.S.); 2 Laboratory of Radiochemistry and Environmental Chemistry, Paul Scherrer Institute, 5232 Villigen-PSI, Switzerland; E-Mails: holger.dorrer@psi.ch (H.D.); konstantin.zhernosekov@gmail.com (K.Z.); andreas.tuerler@psi.ch (A.T.); 3 Laboratory of Radiochemistry and Environmental Chemistry, Department of Chemistry and Biochemistry University of Bern, 3012 Berne, Switzerland; 4 Institut Laue-Langevin, 38000 Grenoble, France; E-Mail: koester@ill.fr; 5 Physics Department, ISOLDE/CERN, 1211 Geneva, Switzerland; E-Mail: karl.johnston@cern.ch; 6 Department of Chemistry and Applied Biosciences, ETH Zurich, 8093 Zurich, Switzerland

**Keywords:** terbium-149, alpha-therapy, folate receptor, DOTA-folate, KB tumors, ^149^Tb-cm09, albumin binder, radionuclide therapy

## Abstract

Terbium-149 is among the most interesting therapeutic nuclides for medical applications. It decays by emission of short-range α-particles (E_α_ = 3.967 MeV) with a half-life of 4.12 h. The goal of this study was to investigate the anticancer efficacy of a ^149^Tb-labeled DOTA-folate conjugate (cm09) using folate receptor (FR)-positive cancer cells *in vitro* and in tumor-bearing mice. ^149^Tb was produced at the ISOLDE facility at CERN. Radiolabeling of cm09 with purified ^149^Tb resulted in a specific activity of ~1.2 MBq/nmol. *In vitro* assays performed with ^149^Tb-cm09 revealed a reduced KB cell viability in a FR-specific and activity concentration-dependent manner. Tumor-bearing mice were injected with saline only (group A) or with ^149^Tb-cm09 (group B: 2.2 MBq; group C: 3.0 MBq). A significant tumor growth delay was found in treated animals resulting in an increased average survival time of mice which received ^149^Tb-cm09 (B: 30.5 d; C: 43 d) compared to untreated controls (A: 21 d). Analysis of blood parameters revealed no signs of acute toxicity to the kidneys or liver in treated mice over the time of investigation. These results demonstrated the potential of folate-based α-radionuclide therapy in tumor-bearing mice.

## 1. Introduction

Targeted radionuclide therapy using β^−^-particle-emitting radionuclides (e.g., ^131^I, ^90^Y, ^177^Lu) of variable energies is employed in clinical routine. For this purpose a variety of tumor-targeted biomolecules have been used, ranging from small-molecular weight compounds (e.g., ^131^I-MIBG), to peptides (e.g., ^177^Lu-DOTATATE) and monoclonal antibodies (^90^Y-Ibritumomab, Zevalin^®^) [1,2]. A promising option for a potential improvement of the therapeutic efficacy of radioendotherapy may be the selection of appropriate radionuclides. α-Particles of medically interesting radionuclides provide a 200- to 1,000-fold higher linear energy transfer (LET) than β^−^-particles [3,4]. Therefore, and because of the much shorter path-length (25–100 μm) of α-particles compared to β^−^-particles (0.05–12 mm), α-particle-emitting nuclides may be interesting for targeted radionuclide therapy of micrometastases or even single cancer cells.

**Figure 1:**
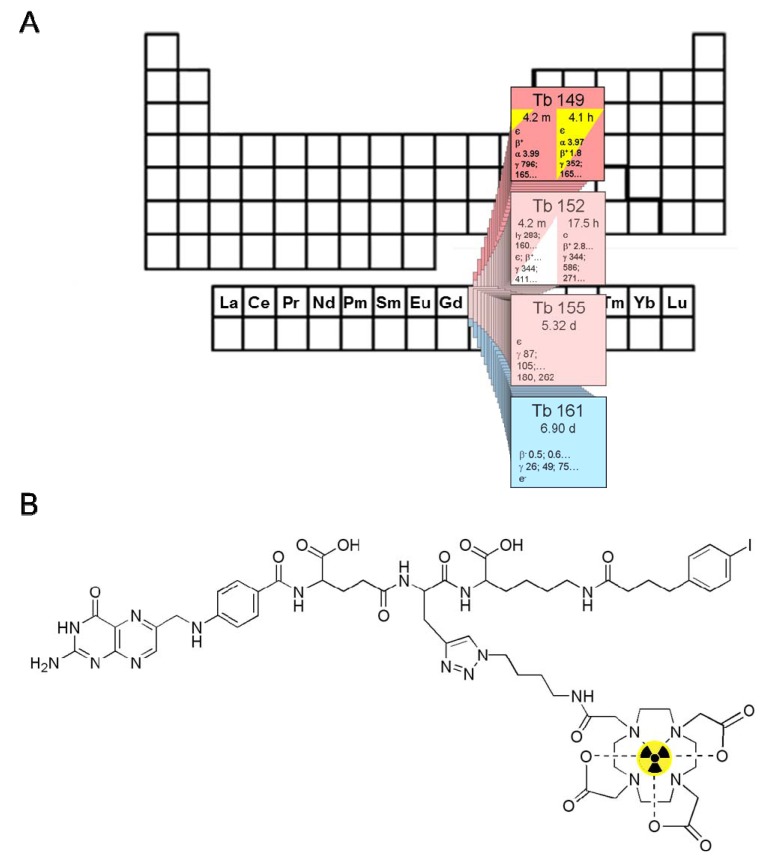
(**A**) The α-particle-emitting ^149^Tb as one of four medically interesting terbium-nuclides which belong to the series of chemical elements called lanthanides (15 elements from La to Lu); (**B**) Chemical structure of the radiolabeled DOTA-folate conjugate (cm09) with an albumin binding entity (speculative coordination sphere of the Tb-DOTA-complex).

Allen *et al.* suggested the radiolanthanide ^149^Tb as the preferred radionuclide for α-radionuclide therapy [3]. A decay scheme with a list of significant emitted radiation of ^149^Tb and its daughter nuclides has been previously published by Beyer *et al.* [5]. ^149^Tb decays with a half-life of 4.12 h by emission of short-range α-particles (E_α_ = 3.967 MeV, I = 16.7%) [4,5]. In addition, it emits γ-rays of an energy (E_γ_ = 165 keV, 26.4%) potentially suitable for single photon emission computed tomography (SPECT) and positrons (E_β+__average_ = 638 keV, 3.8%) which may be detected via positron emission tomography (PET) (Figure 1A) [4,6]. However, there also exists suitable diagnostic matched nuclides such as ^155^Tb (T_1/2_ = 5.32 d, E_γ_= 87 keV, I = 32% and 105 keV, I = 25%) and ^152^Tb (T_1/2_ = 17.5 h, E_β+__average_ = 1.08 MeV, I = 17%) for imaging purposes via SPECT and PET, respectively [7]. Tb can be stably coordinated by macrocyclic chelators (e.g., DOTA) as it has been recently demonstrated using the β^−^-emitting nuclide ^161^Tb [7,8,9]. ^149^Tb is among the most attractive candidates of α-emitting radiometals for targeted radionuclide therapy. Other α-emitters have either a very short half-life (^213^Bi: T_1/2_ = 46 min, ^212^Bi: T_1/2_ = 61 min) or a complicated decay cascade of 4 to 5 α-emissions (^225^Ac: T_1/2_ = 10.0 d, ^227^Th: T_1/2_ = 18.7 d) [4,10,11]. The longer-lived *in vivo* generator ^212^Pb/^212^Bi (T_1/2_ = 10.64 h) might be a more favorable solution but could suffer from release of the ^212^Bi from the DOTA complex [12]. In such cases the decay of the daughter nuclides may occur in non-targeted organs which could cause undesired toxicity to healthy tissue [13]. In spite of the fact that the α-emitting ^211^At (T_1/2_ = 7.21 h) is considered appropriate for medical use with regard to the physical properties, a major impediment to practical applications is the low *in vivo* stability of astatine bonds with aromatic carbon bonds [14]. However, current availability of ^149^Tb is poor due to production routes which are not easily accessible [6]. Potential production routes include irradiation of rare ^152^Gd targets with high energy protons (>50 MeV) or the use of light ions as projectiles of >500 MeV protons for spallation reactions. However, in both cases mass separation is required to avoid radioisotopic impurities.

The aim of the present study was to complement our preliminary *in vivo* results [7] by a further study using ^149^Tb for *in vitro* cell viability assays and *in vivo* using a well-established tumor mouse model. We employed the recently developed DOTA-folate conjugate (herein referred to as cm09, Figure 1B) and human KB cancer cells which express the folate receptor (FR) at high levels. From our previous *in vivo* studies performed with ^177^Lu-cm09, it is known that the novel folate radioconjugate is applicable for preclinical therapy studies in the KB tumor mouse model where it provides an excellent tumor accumulation of radioactivity and as a consequence high antitumor efficacy [15].

## 2. Experimental

### 2.1. Chemicals and Reagents for Production and Purification of ^149^Tb

Gold foils (thickness: 0.1 mm, purity: 99.95%, Goodfellow Cambridge Ltd. Huntingdon, UK) were electrolytically coated with a thin layer of zinc (purity ≥99.9%). The acids in this work (HNO_3_ suprapur and HCl suprapur) were obtained from VWR International GmbH (Dietikon, Switzerland) and diluted with MilliQ water (18.2 MΩ·cm; Millipore AG, Zug, Switzerland). A solution of NH_3_ (25%, suprapur) was obtained from VWR International GmbH. α-Hydroxyisobutyric acid (α-HIBA, purity: 99%), L-lactic acid (C_3_H_6_O_3_, purity ≥99%) and NaOH monohydrate (Traceselect, purity ≥99.9995%) were obtained from Sigma-Aldrich International GmbH (St. Gallen, Switzerland).

### 2.2. Production of ^149^Tb

^149^Tb was produced at the isotope separation online facility ISOLDE (CERN, Geneva, Switzerland) as previously reported [7,16,17]. In brief, a tantalum target (50 g/cm^2^) was irradiated with high-energy (~1.4 GeV) protons. After effusion of the spallation products from the heated target (~2,000 °C) they were ionized by surface ionization and resonant laser ionization. The monocations were extracted from the ion source, accelerated to 50 keV and separated in a magnetic field according to their mass [16,17]. Products of mass number 149 were implanted into a zinc-coated gold foil (70 mm^2^) and shipped to PSI for the purification procedure.

### 2.3. Purification of ^149^Tb

The zinc layer was dissolved in a solution mixture of HNO_3_/NH_4_NO_3_ (0.2 M NO_3_^−^, pH 1, 500 μL) at 50 °C. Isolation of ^149^Tb from isobar and pseudo-isobar impurities and stable Zn was accomplished by cation exchange chromatography. For this purpose a column (5 mm × 35 mm) filled with a strongly acidic macroporous cation exchange resin was employed as recently reported [7,18]. ^149^Tb and ^149^Gd were eluted with α-HIBA (0.13 M) at a flow rate of 0.33 mL/min allowing their separation [7]. Further radioactive impurities and stable Zn remained on the column. Regeneration of the column was carried out using higher concentrated α-HIBA (1.0 M). The collected fractions (330 μL each) which contained ^149^Tb-α-HIBA were acidified by addition of HCl (4 M, 17 μL). The acidic solution was loaded on a second cation exchange chromatography column (4 mm × 4 mm). α-HIBA, NH_4_^+^ and HCl were removed by a washing step using MilliQ water. The elution of ^149^Tb was performed by a solution of L-lactate (0.4 M) previously adjusted to a pH value of 4.7 with sodium hydroxide. The eluted ^149^Tb-fraction was diluted with the 1.7-fold volume of MilliQ water to adjust the osmolarity to a physiological value (~300 mOsm).

### 2.4. Preparation of ^149^Tb-cm09 and Stability in Blood Plasma

Radiolabeling was performed by addition of 18 μL of the DOTA-folate (cm09) stock solution (10 mM, corresp. to 18 nmol of cm09) to the obtained solution of ^149^Tb (25 MBq) in L-lactate (pH 4.7). The reaction mixture was incubated for 10 min at 95 °C. Quality control was performed by HPLC using a C-18 reversed phase column (Xterra MS C-18, 5 μm, 15 cm × 4.6 cm, Waters, Milford, MA, USA). The mobile phase consisted of MilliQ water with 0.1% trifluoroacetic acid (A) and methanol (B) with a linear gradient from 5% to 80% B over 25 min at a flow rate of 1 mL/min. The product (R_t_ = 19.5 min) was obtained with a radiochemical purity of >96%. After addition of Na-DTPA (10 μL, 5 mM, pH 5) the labeling solution (1.0–1.2 MBq/nmol) was directly used for *in vitro* and *in vivo* application. The stability of ^149^Tb-cm09 was investigated by incubation of the radioconjugate (50 μL, ~1 MBq) in human plasma (250 μL) at 37 °C. Aliquots (50 μL) were taken for analysis after 30 min, 1 h and 4 h. The proteins were precipitated by addition of methanol (200 μL) and the supernatants were analyzed using HPLC.

### 2.5. Cell Culture

KB cells (human cervical carcinoma cell line, HeLa subclone; ACC-136) were purchased from the German Collection of Microorganisms and Cell Cultures (DSMZ, Braunschweig, Germany). Cells were cultured as monolayers at 37 °C in a humidified atmosphere containing 5% CO_2_. Importantly KB cells were cultured in a folate-free special cell culture medium, FFRPMI (modified RPMI, without folic acid, vitamin B_12_ and phenol red; Cell Culture Technologies GmbH, Gravesano/Lugano, Switzerland). FFRPMI media was supplemented with 10% heat-inactivated fetal calf serum (FCS), L-glutamine and antibiotics (penicillin/streptomycin/fungizone). Routine culture treatment was performed twice a week.

### 2.6. In Vitro Cell Viability Studies

Inhibition of KB cell viability was investigated using a 3-(4,5-dimethylthiazol-2-yl)-2,5-diphenyltetrazolium bromide (MTT) assay [19]. The cells were harvested and seeded in 96-well plates at 2.5 × 10^3^ cells per well in a final volume of 200 μL FFRPMI medium with supplements. After 24 h incubation for cell adhesion, the medium was removed and the cells were incubated with ^149^Tb-cm09 (0.05–500 kBq in 200 μL medium/well) alone or in combination with excess folic acid (200 nM) for 4 h at 37 °C. Cell incubation with FFRPMI medium only was performed as a control experiment. After incubation, the supernatants were removed and KB cells were washed with PBS (200 μL/well) before addition of supplemented FFRPMI medium (200 μL/well). Cells were then allowed to grow for 4 days before analysis as previously reported [20]. After addition of 30 μL of an MTT solution (5 mg/mL) to each well, the well-plates were incubated for an additional 4 h at 37 °C. The medium was removed and the dark-violet formazan crystals were dissolved in dimethyl sulfoxide. The absorbance was determined at 560 nm using a microplate reader (Victor X3, Perkin Elmer, Waltham, MA, USA). To quantify cell viability, the ratio of the absorbance of the test samples to the absorbance of control cell samples (=100% viability) was calculated using Microsoft Excel software (Microsoft Corp. Redmond, WA, USA).

### 2.7. In Vivo Therapy Studies

*In vivo* experiments were approved by the local veterinary department and conducted in accordance with the Swiss law of animal protection. Four to five-week-old female, athymic nude mice (CD-1 Foxn-1/nu) were purchased from Charles River Laboratories (Sulzfeld, Germany). They were fed with a folate-deficient rodent diet (ssniff Spezialdiäten GmbH, Soest, Germany) starting 5 days prior to tumor cell inoculation [21]. Endpoint criteria were defined as weight loss of >15% of the initial body weight (at day 0), tumor volume >1,000 mm^3^, ulceration or bleeding of the tumor xenograft or abnormal behavior indicating pain or unease of the animal.

The therapy study was performed according to the injection protocol shown in Table 1. Twelve mice were subcutaneously inoculated with 5 × 10^6^ KB tumor cells (suspended in 100 μL PBS) as previously reported 4 days before injection of the radioactive folate conjugate [15].

**Table 1:** Injection protocol of the *in vivo* therapy study.

	Number of mice [n]	Injection solution	Injected radioactivity [MBq]
Group A	4	L-lactate solution	-
Group B	4	^149^Tb-cm09 in L-lactate solution	2.2
Group C	4	^149^Tb-cm09 in L-lactate solution	3.0

At the time of treatment the tumors reached a volume 60–80 mm^3^. Mice of group A were intravenously injected with only L-lactate solution whereas mice of group B and C received ^149^Tb-cm09 (2.2 MBq and 3.0 MBq, respectively) in the same L-lactate solution. The amount of injected activity was the highest possible based on the availability of the nuclide sufficient to inject 4 mice. After start of the therapy mice were weighed three times a week over 35 days and tumor volumes were monitored by measuring two perpendicular diameters with a caliper and calculated according to the equation: V = 0.5 × L × W^2^ (L is the length (large diameter) and W is the width (small diameter) of the tumor). Mice were removed from the study if one or several of the predefined endpoint criteria were reached which required euthanasia.

The therapeutic efficacy was expressed as the percentage of tumor growth inhibition (%TGI) calculated according to the formula: %TGI = 100 − (RTV_T_/RTV_U_ × 100), where RTV_T_ is the mean relative tumor volume of treated mice (groups B or C), and RTV_U_ is the mean relative tumor volume of untreated mice (group A) determined at day 21 when the first control mouse was euthanized. The tumor growth delay index (TGDI) was calculated according to the formula: TGDI = TGD_T_/TGD_U_. It was defined as the mean tumor growth delay ratio of treated (TGD_T_) and untreated animals (TGD_U_) which was required to increase the RTV 5-fold [22].

### 2.8. Determination of Plasma Parameters

Plasma parameters such as blood urea nitrogen (BUN), alkaline phosphatase (ALP), and total bilirubin (TBIL) were measured from all mice at day 14 after start of the therapy and before euthanasia of each mouse. BUN is a common parameter to determine potential damage to the kidneys whereas increased ALP and TBIL could be an indication for impaired liver function. Plasma samples were prepared by centrifugation of blood samples (150–200 μL per mouse) drawn from the sublingual vein of each mouse and collected in heparinized vials (Microvette, 200 mL Sarstedt, Nümbrecht, Germany). For each parameter a plasma volume of 10 μL was required for the analysis using a Fuji Dri-Chem 40000i analyzer (Polymed Medical Center AG, Glattbrugg, Switzerland).

### 2.9. Dosimetric Calculations

To estimate the equivalent absorbed dose for ^149^Tb-cm09 in KB tumor xenografts, biodistribution data obtained with ^161^Tb-cm09 were used. Based on these data the following calculations were made: (i) the cumulative radioactivity was calculated from integrated AUCs (MBq·s) of biodistribution data expressed in non decay-corrected percent injected activity [%IA] per tumor mass (assumed as 100 mg, due to an approximate calculated volume of the tumors at the start of the therapy); (ii) the adsorbed radiation dose in tumor xenografts was assessed for a sphere of 100 mg using the Unit Density Sphere Model from RADAR [23]; (iii) The absorbed dose (mGy/MBq) was calculated by multiplying the AUC (s; normalized to 1 MBq ID) with the S-value (mGy/MBq·s); and (iv) the dose (mGy) was calculated by multiplying the absorbed dose (mGy/MBq) with the amount of injected radioactivity.

### 2.10. Statistical Significance

Significance of the survival time and tumor growth delay was calculated by the performance of the *t*-test (Microsoft Excel). All analyses were two-tailed and considered as type 3 (two sample unequal variance). A *p*-value < 0.05 was considered statistically significant.

## 3. Results and Discussion

### 3.1. Production and Purification of ^149^Tb

**Figure 2:**
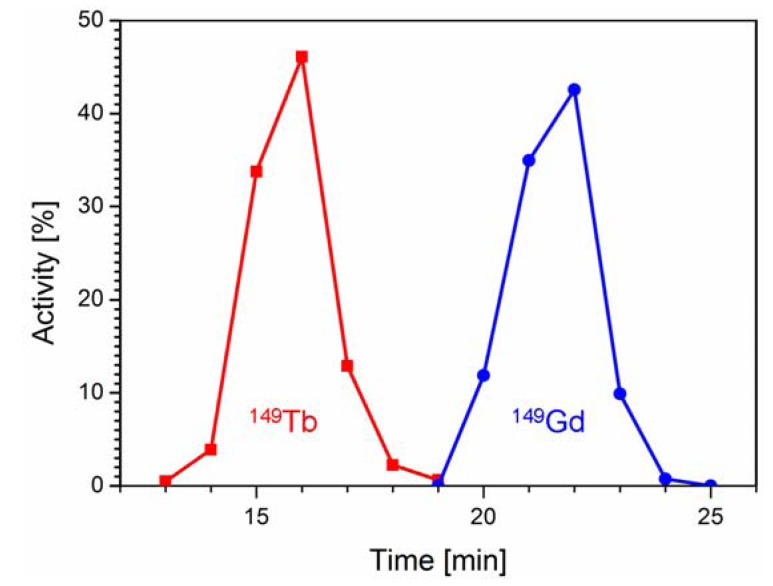
Elution profile of chromatographic column I showing separation of ^149^Tb (■, red) from ^149^Gd (●, blue).

^149^Tb was produced by proton-induced spallation of tantalum targets at ISOLDE/CERN on a zinc-covered gold foil containing isobar and pseudo-isobar nuclides of mass number 149. Upon arrival at PSI (~4 h after the end of collection) ~40 MBq ^149^Tb, ~5 MBq ^149^Gd, ~0.4 MBq ^145^Eu (from α-decay of ^149^Tb), ~70 MBq ^133m^Ce, ~15 MBq ^133^Ce and ~350 MBq ^133^La were detected. The mass 133 pseudo-isobars appear at mass 149 as monoxide molecular ions. The radioactive solution which was obtained after dissolution of the zinc-layer was loaded on column I for chromatographic separation of ^149^Tb. The elution was accomplished with a diluted solution of α-HIBA within only 25 min. The ^149^Tb (~29 MBq, corresponding to 73% of total radioactivity) was isolated in a volume of 1.65 mL free of radioactive impurities. Under these isocratic elution conditions an excellent separation of ^149^Tb and ^149^Gd was achieved (Figure 2). Further radiolanthanide impurities such as ^145^Eu, ^133m^Ce, ^133^Ce and ^133^La as well as stable Zn were retained on the chromatographic column I and eluted afterwards using a higher concentrated α-HIBA solution for regeneration of the column.

After concentration of the ^149^Tb-solution by adsorption at the cation exchanger of the second column, the product was obtained within 25 min as ^149^Tb-L-lactate (0.4 M, pH 4.7) in a small volume of only 225 μL. Addition of 375 μL MilliQ water resulted in a solution of 0.15 M sodium L-lactate with a physiological osmolarity of ~300 mOsm. Determination of radioactivity revealed a total yield of ~25 MBq ^149^Tb containing ~88 kBq ^149^Gd—which decays via electron capture and γ-ray emission—as the only detectable radionuclide impurity generated by the decay of ^149^Tb after separation on column I. The total yield of the separation process was ~63% (corresponding to a ~94% decay-corrected yield) and the whole isolation process including measurement of the single elution fractions was accomplished within 145 min. The L-lactate-based physiological formulation was suitable not only for the direct performance of radiolabeling of cm09 but allowed even *in vitro* and *in vivo* application without further purification steps.

In comparison to our previous study [7] where we used ^149^Tb for radiolabeling directly after elution from the first column in the α-HIBA solution (~0.15 M), the purification process was optimized in the present study. The advantages were two-fold: Firstly, using the second chromatographic column allowed concentration of the radioactivity in a smaller volume which was favorable for subsequent radiolabeling steps and *in vivo* application. Secondly, the elution was carried out with a solution of L-lactate providing ^149^Tb in a more physiological solution compared to the α-HIBA solution which was employed in our previous studies.

### 3.2. Radiosynthesis of ^149^Tb-cm09 and Stability in Blood Plasma

Radiolabeling of cm09 (18 nmol) with ^149^Tb (25 MBq) resulted in a product peak of the radiolabeled product with a retention time (R_t_) of 19.5 min. The chromatogram was equal to what was previously obtained with the ^161^Tb-labeled match (^161^Tb-cm09) [9]. On the other hand injection of free ^149/161^Tb(III) was eluted almost with the front (R_t_ = 3 min). These findings confirmed the identity of the product peak as ^149^Tb-cm09 which was obtained with a radiochemical purity of >96% with only insignificant traces of free ^149^Tb(III).

Incubation of ^149^Tb-cm09 (1.0–1.2 MBq/nmol) in blood plasma at 37 °C revealed high stability (>98%) of the folate radioconjguate over at least 4 hours. These findings were expected as it was previously shown that ^161^Tb-cm09 was completely stable in human blood plasma over several days [7,9].

### 3.3. In Vitro Application of ^149^Tb-cm09

**Figure 3:**
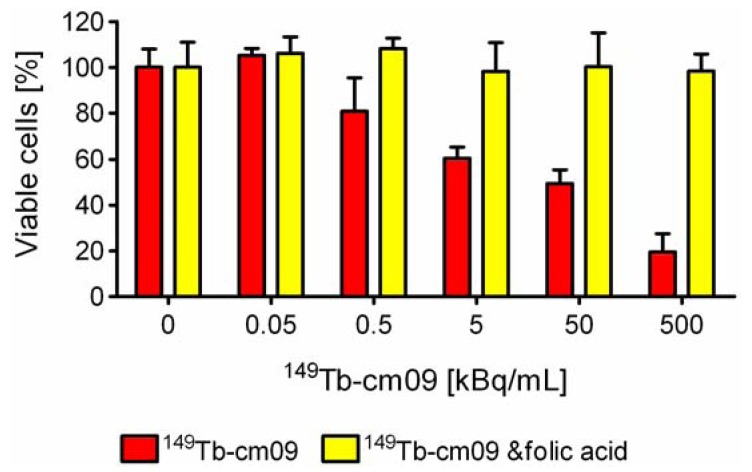
*In vitro* viability of KB tumor cells is reduced upon exposure to increasing radioactivity concentrations of ^149^Tb-cm09 (red bars). Incubation of cells with excess folic acid to block FRs protected KB cells from the ^149^Tb-cm09-induced inhibitory effect (yellow bars). Exposure of cells to unlabeled cm09 (100 nM to 10 μM) had no effect on cell growth (data not shown).

*In vitro* cell viability assays were performed to investigate the effect of ^149^Tb-cm09. FR-positive KB tumor cells were incubated with increasing radioactivity concentrations of ^149^Tb-cm09. It was found that the viability of the KB cells was inhibited in an activity-dependent manner (Figure 3). Application of a low radioactivity concentration of ^149^Tb-cm09 (0.5 kBq/mL) resulted in an only 20%-reduction of the tumor cell viability. However, a 1,000-fold higher radioactivity concentration (500 kBq/mL) resulted in an almost complete loss of KB cell viability. Addition of excess folic acid to block cell surface exposed FRs abolished the effect of ^149^Tb-cm09 completely even at high radioactivity concentrations. Under these conditions tumor cells grew normally and comparably to untreated control cells (Figure 3). These findings clearly indicated a FR-specific effect of ^149^Tb-cm09.

### 3.4. Therapy Study in Tumor-Bearing Mice Using ^149^Tb-cm09

**Figure 4:**
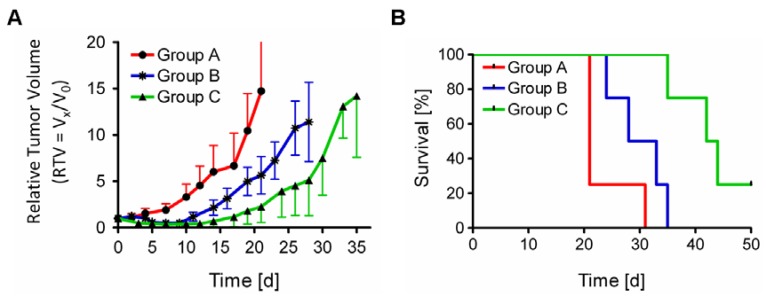
Results of FR-targeted radionuclide α-therapy using ^149^Tb-cm09 in KB tumor-bearing mice. (**A**) Graph of the average relative tumor volume (RTV) of mice from each group (*n* = 4) during the time period when at least 3 mice were still alive; (**B**) Survival curves of mice from each group (group A: 21 d; group B: 30.5 d and group C: 43 d).

The therapy study in KB tumor-bearing nude mice showed significant tumor growth delay (TGD) in ^149^Tb-cm09 treated mice compared to untreated control mice (Figure 4A). The tumor growth delay index for a 5-fold increased tumor volume compared to the tumor volume at the beginning of the study (TGDI_5_) reached a value of 1.5 (*p* = 0.04) for mice of group B which were treated with 2.2 MBq ^149^Tb-cm09. This means a 1.5-fold higher value than for untreated control mice (TGDI_5_ = 1). In mice of group C, which received 3.0 MBq of ^149^Tb-cm09, the TGDI_5_ was even 2.0-fold (*p* = 0.01) increased compared to the TGDI_5_ of untreated controls. At day 21 when the first control mouse had to be euthanized due to an oversized tumor the average relative tumor volume (RTV) was significantly smaller in treated mice of group B (*p* = 0.01) and group C (*p* = 0.001) compared to the tumor volume of untreated control mice (group A). At this time point of the study tumor growth was inhibited by 62% (group B) and 85% (group C), respectively, indicating a dose-dependent therapeutic efficacy of ^149^Tb-cm09. The average survival time was increased by 45% (30.5 d, *p* = 0.01) in mice of group B and by 105% (43 d, *p* = 0.004) in mice of group C compared to control mice (group A, 21 d) (Figure 4B).

Analysis of blood plasma parameters (BUN, ALP and TBIL) did not reveal significant changes (*p* > 0.05) among control mice and mice treated with ^149^Tb-cm09 (Table 2). This analysis indicated unimpaired renal and hepatobiliar function. Hence, it can be concluded that acute radiotoxic effects to the kidneys and the liver were not experienced by FR-targeted α-radionuclide therapy over the whole time of this study which lasted for 35 days. More detailed studies to investigate effects of ^149^Tb-cm09 on kidney function will be necessary since undesired side-effects on the long-term cannot be excluded based on the examinations performed herein.

**Table 2:** Results of plasma analysis at day 14 and before terminal (in parentheses); (BUN = blood urea nitrogen, ALP = alkaline phosphatase, TBIL = total bilirubin).

	BUN [mmol/L]	ALP [U/L]	TBIL [μmol/L)
Group A	7.0 ± 1.8 (4.1 ± 0.5)	77 ± 10 (99 ± 16)	6.5 ± 2.5 (6.3 ± 1.0)
Group B	4.6 ± 0.6 (4.7 ± 0.4)	67 ± 5 (119 ± 7)	7.6 ± 4.7 (6.0 ± 1.0)
Group C	5.2 ± 1.3 (5.9 ± 1.4)	75 ± 14 (73 ± 21)	7.0 ± 0.8 (5.0 ± 1.0)

Overall the present results outperformed our previous data which were obtained upon administration of two injections of ^149^Tb-cm09 at low quantities of radioactivity (1.1 MBq and 1.3 MBq, respectively) [7]. Compared to our previous therapy study, the present study design differed in that the mice received the whole amount of radioactivity in a single injection of either 2.2 MBq ^149^Tb-cm09 (group B) or 3.0 MBq ^149^Tb-cm09 (group C).

Beyer *et al.* conducted an experiment where ^149^Tb-labeled rituximab (5.5 MBq per mouse) was investigated in a leukemia animal model using SCID mice with an intravenous graft of Daudi cells [5]. Their aim was to examine the efficacy of ^149^Tb-rituximab to specifically kill circulating single cancer cells or small cell clusters *in vivo*. The therapy was started within 3 days upon intravenous xenografting of a lethal number of Daudi cells when most of the tumor cells were expected to be still in circulation [5]. Whereas untreated control mice had to be euthanized within the first 37 days a tumor-free survival was found for over 120 d in almost 90% of the ^149^Tb-rituximab treated animals. Since application of the same amount of unlabeled rituximab did not show a therapeutic effect, the favorable outcome of this study could be ascribed to the α-radionuclide therapy [5]. In the present study injection of ^149^Tb-cm09 reduced tumor growth of solid KB xenografts and increased the survival time significantly in mice of both treated groups (B and C) compared to untreated control mice (group A). These excellent results complement those of Beyer *et al.* by demonstrating the therapeutic potential of ^149^Tb. However, our study design differed from that of Beyer *et al.* in three respects. (i) Instead of using an established antibody, we employed a small-molecular weight folate conjugate (cm09) as a targeting agent which previously proved the promising potential to be used for therapeutic purposes [15]; (ii) The amount of injected radioactivity was 2.2 MBq and 3.0 MBq, respectively, in our study compared to 5.5 MBq which were used in the study of Beyer *et al.* [5]; (iii) Finally the tumor mouse model which was used in our study was based on solid tumor xenografts of a human cervical cancer cell line while Beyer *et al.* used a tumor mouse model with circulating leukemia cells.

### 3.5. Dosimetric Calculations

In order to obtain an idea about the radioactive dose burden of ^149^Tb-cm09 to KB tumor xenografts a dose estimation was made while taking only the self-radiation dose into account. According to the AUC obtained from tissue distribution data in mice injected with ^161^Tb-cm09 [7] and the S-value of ^149^Tb listed for a sphere of 100 mg an absorbed dose of 8.67 Gy/MBq was estimated for KB tumor xenografts. This resulted in an absorbed dose of ~19 Gy (group B) and ~26 Gy (group C) in tumors upon a single injection of 2.2 MBq and 3.0 MBq of ^149^Tb-cm09, respectively.

Recently, we peformed a preclinical therapy study with 10 MBq of ^161^Tb-cm09 using the same KB tumor mouse model [9]. In that case the dose to the tumor was calculated to a value of ~33 Gy and the corresponding TGDI_5_ reached a value of ~2.2. In the present study the TGDI_5_ for ^149^Tb-cm09 was ~1.6 (2.2 MBq, ~19 Gy) and ~2.0 (3.0 MBq, ~26 Gy) indicating a slightly improved effect of the α-radionuclide therapy. However, for an exact comparison of α- and β^−^/Auger-radionuclide therapy it would be necessary to perform a site-by-site study using ^149^Tb-folate and ^161^Tb-folate with an activity that results in the same calculated dose to the tumor.

## 4. Conclusions

In this study the potential of α-radionuclide therapy in general and of ^149^Tb in particular was demonstrated using a folate-based biomolecule. Application of ^149^Tb-cm09 was well-tolerated in mice and treated KB tumor xenografts efficiently. The experiments revealed significant tumor growth delay and an increased survival time of mice treated with ^149^Tb-cm09 compared to untreated control mice. However, further studies will be required to investigate potential advantages of FR-targeted α-radionuclide therapy over β^−^-radionuclide therapy with regard to tumor response and potential damage to the kidneys.
